# High-Fat Diet-Induced Blood–Brain Barrier Dysfunction: Impact on Allodynia and Motor Coordination in Rats

**DOI:** 10.3390/ijms252011218

**Published:** 2024-10-18

**Authors:** Laura M. Ubaldo-Reyes, Estefania Espitia-Bautista, Antonio Barajas-Martínez, Ricardo Martínez-Tapia, Verónica Rodríguez-Mata, Roxana Noriega-Navarro, Rene Escalona, Jesús Castillo-Hernández, Armando Pérez-Torres, Luz Navarro

**Affiliations:** 1 Department of Anatomy, Facultad de Medicina, Universidad Nacional Autónoma de México, Mexico City 04510, Mexico; 2 Laboratory of Molecular Neurophysiology, National Institute of Psychiatry Ramón de la Fuente, Mexico City 14370, Mexico; estefaneb@hotmail.com; 3 Center for Complexity Science, Universidad Nacional Autónoma de México, Mexico City 04510, Mexico; antonio.barajas@c3.unam.mx; 4 Department of Physiology, Facultad de Medicina, Universidad Nacional Autónoma de México, Mexico City 04510, Mexico; ricardo.mtapia@gmail.com (R.M.-T.); roxnn77@gmail.com (R.N.-N.); lnavarro@unam.mx (L.N.); 5 Department of Histology, Facultad de Medicina, Universidad Nacional Autónoma de México, Mexico City 04510, Mexico; verohistologabct@hotmail.com (V.R.-M.); armandop@unam.mx (A.P.-T.); 6 Laboratory of Embryology and Genetics, Departamento de Embriología y Genética, Facultad de Medicina, Universidad Nacional Autónoma de México, Mexico City 04510, Mexico; escalonarj@facmed.unam.mx; 7 Multidisciplinary Academic Unit Middle Zone, Autonomous University of San Luis Potosí, San Luis Potosí 79615, Mexico; jesus.castillo@uaslp.mx

**Keywords:** neuroimmunology, nociception, neuronal integrity, motor impairment, high-fat diet

## Abstract

The associations among increased pain sensitivity, obesity, and systemic inflammation have not been described as related to BBB dysfunctions. To analyze the metabolic, behavioral, and inflammatory effects of a high-fat diet (HFD) and ultrastructural modifications in brain regions, we used an in vivo experimental model. Adult male Wistar rats were randomly assigned to one of two conditions, an ad libitum control group or an HFD (60%)-fed group, for eight weeks. At the end of the protocol, glucose and insulin tolerance tests were performed. Additionally, we analyzed the response to a normally innocuous mechanical stimulus and changes in motor coordination. At the end of the protocol, HFD-fed rats presented increased HOMA–IR and metabolic syndrome (MetS) prevalence. HFD-fed rats also developed an increased nociceptive response to mechanical stimuli and neurological injury, resulting in impaired motor function. Hypothalamus and cerebellum neurons from HFD-fed rats presented with nuclear swelling, an absence of nucleoli, and karyolysis. These results reveal that HFD consumption affects vital brain structures such as the cerebellum, hippocampus, and hypothalamus. This, in turn, could be producing neuronal damage, impairing cellular communication, and consequently altering motricity and pain sensitivity. Although direct evidence of a causal link between BBB dysfunction and sensory-motor changes was not observed, understanding the association uncovered in this study could lead to targeted therapeutic strategies.

## 1. Introduction

Obesity leads to a wide range of complications, including the development of metabolic syndrome (MetS), which is characterized by abdominal obesity, elevated triglycerides, low HDL cholesterol, hypertension, disrupted glucose homeostasis, and insulin resistance [1]. The pathophysiology of MetS is driven by a positive energy balance, leading to adipose tissue hyperplasia and hypertrophy, along with altered leptin signaling [2]. A high-fat diet (HFD) is one of the most commonly used methods to induce obesity in animal models [3,4,5,6]. Chronic consumption of an HFD has been linked not only to metabolic dysregulation but also to cognitive impairments [7], neuroinflammation [8], and the blood–brain barrier (BBB) dysfunction [9,10,11]. The integrity of the BBB is crucial for maintaining brain homeostasis; its disruption can lead to altered permeability and dysregulated signaling in the central nervous system (CNS). BBB dysfunction in obesity, characterized by altered expression of proteins such as connexin-43 [12] and tight junction proteins like occludin [13], has been associated with neuronal damage and behavioral changes. Specifically, studies have shown decreased expression of tight junction proteins in the hypothalamus, which compromises the barrier’s protective function [14]. Additionally, enhanced permeability has been observed in hypothalamic regions, such as the arcuate nucleus, using Evans blue dye diffusion [15]. In response to this BBB dysfunction, an increase in glial fibrillary acidic protein (GFAP) immunoreactivity has been detected in the hypothalamus and arcuate nucleus, indicative of gliosis [16,17]. Moreover, ultrastructural changes, such as vacuolar degeneration in the hippocampus [18] and damage to Purkinje and granular cells in the cerebellum [19], highlight the neuroanatomical consequences of prolonged HFD exposure.

The link between obesity-induced systemic inflammation and BBB dysfunction suggests that these processes may also contribute to altered sensory [20] and motor functions [21]. Specifically, it was proposed that the combination of chronic inflammation, metabolic stress, and BBB impairment affects regions involved in pain processing and motor coordination, such as the hippocampus and cerebellum. Recent studies have indicated that animals fed an HFD exhibit increased pain sensitivity as early as 8 weeks, with these effects persisting for up to 12 weeks [22,23]. Additionally, motor coordination impairments have been observed at various time points, including 4, 12, and 22 weeks [24,25,26]. These functional alterations in both sensory and motor domains highlight the potential role of BBB dysfunction in mediating the effects of obesity on the brain. This study aimed to investigate the relationship between ultrastructural changes in the BBB and neuroanatomical regions associated with pain perception and motor function, particularly the cerebellum and hippocampus, in a model of obesity induced by HFD. Furthermore, the hypothalamus was examined due to its known role in MetS, providing a comprehensive assessment of how BBB disruption may contribute to the behavioral and physiological changes observed in this model.

## 2. Results

### 2.1. Establishing a Model of Obesity

#### 2.1.1. Body Weight, Food Consumption, and Adiposity

**Figure 1:**
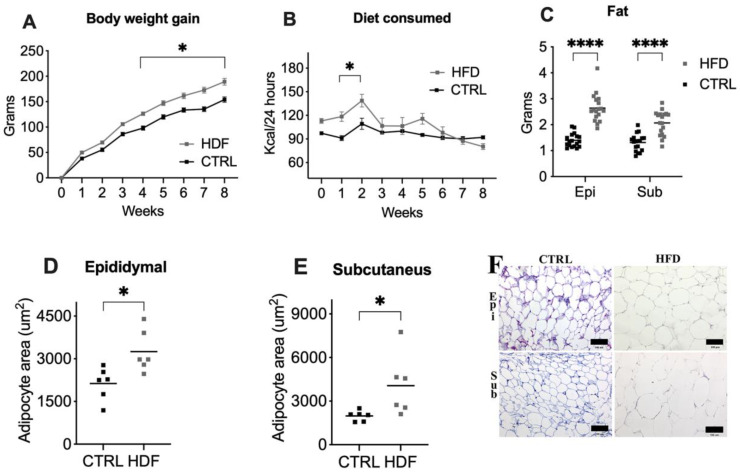
Model of obesity induced by an eight-week HFD. (A) Weekly body weight gain; (**B**) diet consumed; (**C**) fat accumulation. Epididymal Epi, Subcutaneous Sub The data are expressed as the means ± SEMs (*n* = 18 rats). (**D**,**E**) Adipocyte areas of the epididymal and subcutaneous tissues, respectively. The data are expressed as the means ± SEMs (*n* = 6 rats). The control group (CTRL) is represented by black symbols, and the HFD-fed group is represented by gray symbols. Asterisks indicate significant differences compared with the control group (* *p* < 0.05, **** *p* < 0.0001). (**F**) Representative microphotograph (**C**) of adipose tissue (4 µm) with hematoxylin–eosin-stained paraffin sections. Black bars represent 100 µm.

To evaluate the effects of the HFD in rats, weight gain and caloric intake were recorded over eight weeks. The pattern of obesity induced by an HFD was compared via Repeated Measure ANOVA (RM ANOVA), which revealed a significant week × diet interaction effect (F(8272) = 8.36, *p* < 0.0001), and we observed that HFD-fed rats gained more weight (126.3 ± 3.36 SEM) than did those fed a standard chow diet (98.02 ± 4.19 SEM) at week 4, and significant differences (*p* < 0.0001) were maintained until the end of the protocol (Figure 1A). However, the HFD-fed animals consumed more calories in the first two weeks than the CTRL animals did (Figure 1B), and the RM ANOVA revealed a significant week × diet interaction (F(8272) = 4.23, *p* = 0.0001).

The proportion of adipose tissue in both groups was measured to determine whether the weight gain was due to fat mass expansion. Compared with those in the CTRL group, HFD-fed animals accumulated more epididymal (t = 8.84, df = 34, *p* < 0.0001) and subcutaneous (t = 5.62, df = 34, *p* = 0.0001) fat, as measured by weighing dissected fat pads after necropsy (Figure 1C). The size of the adipocytes increased (hypertrophy) in both the epididymal (t = 2.94, df = 10, *p* < 0.0147) and subcutaneous (t = 2.39, df = 10, *p* = 0.0379) tissues of the HFD-fed animals (Figure 1D–F). These findings demonstrate that the increased weight gain observed in HFD-fed rats is attributed to increased accumulation of epididymal and subcutaneous fat, alongside adipocyte hypertrophy.

#### 2.1.2. Effects of an 8-Week HFD on Somatometric and Metabolic Modifications

Somatometric and metabolic parameters in both groups were summarized. As previously mentioned, HFD consumption induced increased body mass due to increased fat depots; in addition, HFD-fed rats presented an increased mass–body length ratio (determined by the body mass index (BMI) and Lee index). Moreover, HFD-fed animals presented an increased waist circumference, which is noteworthy because central obesity is a crucial feature of MetS in humans. In addition to increased fasting plasma glucose (FPG) concentrations, plasma analysis revealed that HFD-fed rats presented increased plasma leptin and insulin concentrations (Table 1). Moreover, HFD-fed animals presented an increased homeostatic model assessment–insulin resistance (HOMA–IR). Eight weeks of HFD consumption led to somatometric and metabolic changes, marked by increased body mass, central obesity, elevated plasma leptin and insulin levels, and a high HOMA–IR, reflecting key features associated with MetS.

**Figure 2:**
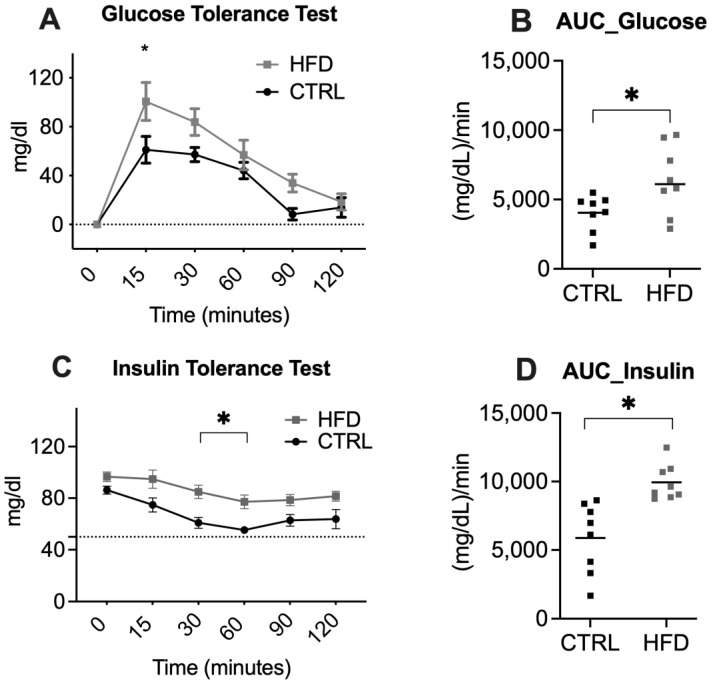
Tolerance tests for glucose and insulin. (**A**) Intraperitoneal glucose tolerance test; (**B**) area under the curve for glucose levels. (**C**) Insulin tolerance test; (**D**) area under the curve for insulin levels. The control group (CTRL) is represented by black symbols, and the HFD-fed group is represented by gray symbols. The data are expressed as the means ± SEMs (*n* = 8 rats). Asterisks indicate significant differences compared with the control group (*p* < 0.05). The horizontal gray line over the X-axis indicates baseline 0.

FPG levels were measured to identify the presence of metabolic alterations, and an intraperitoneal glucose tolerance test (IPGTT) and insulin TT (ITT) were performed. The HFD-fed animals exhibited impaired glucose tolerance via the IPGTT (Figure 2A,B), and the RM ANOVA revealed significant differences over time (F(5,70) = 38.11, *p* < 0.0001) and between groups (F(1,14) = 5.81, *p* = 0.03) but not in the interaction; the area under the curve (AUC) for impaired glucose tolerance was greater for the HFD-fed group than for the CTRL group (t = 2.593, df = 7, *p* = 0.0358). In the ITT (Figure 2C,D), the RM ANOVA revealed significant differences over time (F(2.797,34.68) = 15.03, *p* < 0.001) and between groups (F(1,14) = 16.60, *p* = 0.0011) but not in the interaction; the AUC was greater for the HFD-fed group than for the CTRL group (t = 2.706, df = 14, *p* = 0.0170). These results suggest that HFD treatment induced impaired glucose metabolism and was associated with typical signs of insulin resistance and MetS.

### 2.2. Behavioral Tests and Damage Induced by an HFD

**Figure 3:**
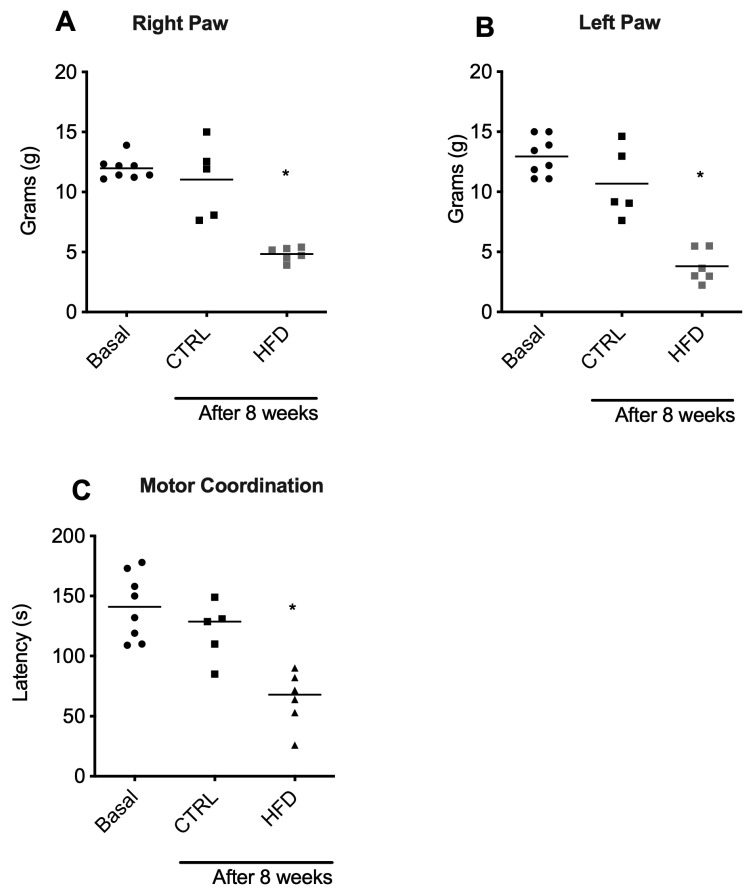
Changes in nociceptive behavior and motor coordination induced by an eight-week HFD. Mechanical allodynia was determined with von Frey filaments via the up–down method in the right paw (**A**) and the left paw (**B**). (**C**) Latency to fall during rotarod testing. The control group is represented by black symbols, and the HFD-fed group is represented by gray symbols. The data are expressed as the means ± SEMs (*n* = 6 rats). Basal *, control and after 8 weeks with a chow diet; *p* < 0.05 vs. the control and HFD-fed groups after 8 weeks. (PWT) 50% paw withdrawal threshold.

Behavioral response assessment was performed via tests of pain and motor coordination. When mechanical allodynia via von Frey filaments was evaluated, rats fed an HFD for 8 weeks (obese rats) presented a significant (*p* < 0.05) decrease in the 50% paw withdrawal threshold (PWT) on both hind paws compared with those in the CTRL group (Figure 3A,B), indicating that rats fed an HFD for 8 weeks developed an increased nociceptive response to mechanical stimuli. When motor coordination was assessed via the rotarod with accelerating speed, obese rats also presented a significant (*p* < 0.05) decrease in latency to fall compared with that of CTRL (Figure 3C), indicating that the motor capacity of obese rats was impaired.

### 2.3. Evaluating BBB Integrity

**Figure 4:**
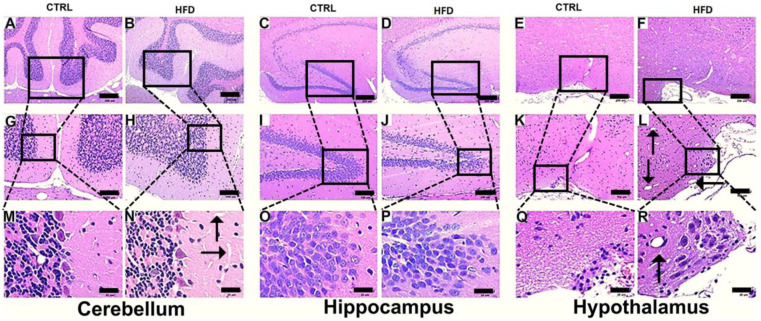
Photomicrographs of different regions of the CNS from control and HFD-fed rats. Compared with those of control rats (**A**,**G**,**M**), the cerebellar cortex of HFD-fed rats presented an increased number of cells in the molecular layer, probably corresponding to stellate cells and basket cells, and a more prominent blood capillary network [(**B**,**H**,**N**) more prominent blood capillary network [(N) (arrows)]. In the hippocampus, the nuclei of neurons from the superior and inferior blades and hinge of the dentate gyrus (DG) in HFD-fed rats (**D**,**J**,**P**) seem to have more compacted chromatin, and the neuropil was observed to be smaller; note the increased cellularity of the GD and normal eosinophilia of the neuropil in controls (**C**,**I**,**O**). In the hypothalamus anterior to the tuberal region, more dilated vessels were observed in the HFD-fed rats (**F**,**L**,**R**) more dilated vessels were observed in the HFD-fed rats [(**L**,**R**) (arrows)], and many cell nuclei were more hyperchromatic than they were in the control animals (**E**,**K**,**Q**). H&E staining. Black bars A to F represent 200 μm, G to L 100 µm, and M to R 25 µm.

Initially, to study brain tissue damage and assess morphological changes in cells in each neuroanatomical region, processed tissues were analyzed via hematoxylin–eosin (H&E) staining. In HFD-fed mice, the cerebellar cortex (Figure 4B,H,N) presented an increased number of cells in the molecular layer. In the hippocampus (Figure 4D,J,P), the nuclei of neurons from the superior and inferior blades and the hinge of the dentate gyrus (DG) seemed to have more compacted chromatin and smaller neuropils. In the hypothalamus (Figure 4F,L,R), dilated vessels and many cell nuclei were more hyperchromatic in the HFD-fed group than in the CTRL group. HFD consumption led to notable morphological changes across multiple neuroanatomical regions, supporting the study’s aim to investigate brain tissue damage induced by dietary factors.

**Figure 5:**
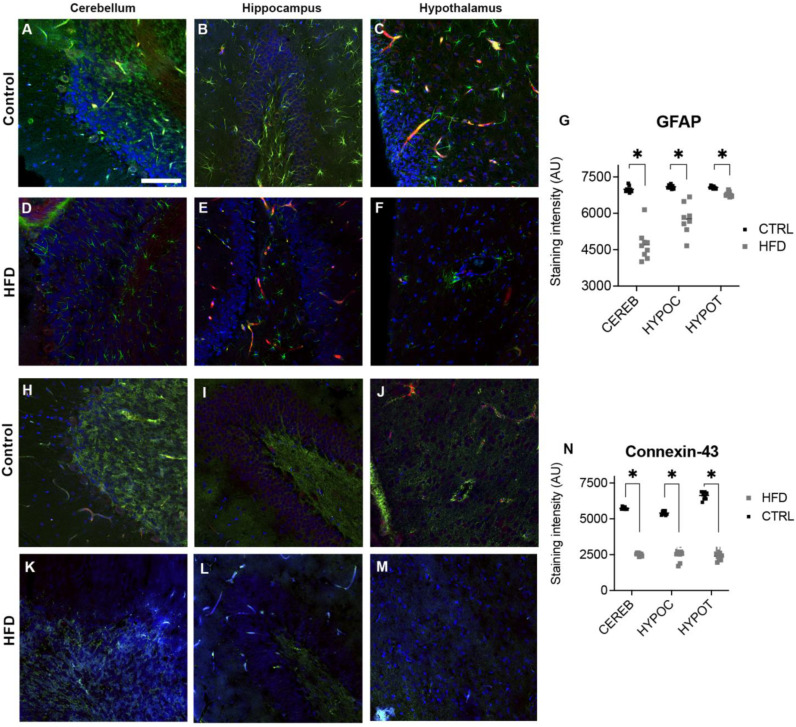
GFAP (A–G) and CX43 (H–N) expression in different regions of the CNS. The figure shows different CNS regions: cerebellar GFAP (**A**,**D**) and Cx-43 (**H**,**K**), hippocampal GFAP (**B**,**E**) and Cx-43 (**I**,**L**), and hypothalamic GFAP (**C**,**F**) and Cx-43 (**J**,**M**) in the control group (**A**–**C**,**H**–**J**) and HFD group (**D**–**F**,**K**–**M**). GFAP (**G**) and Cx-43 (**N**) intensity analysis. (*n* = 3 rats with 3 slices in each one). The control group is represented by black symbols, and the HFD-fed group is represented by gray symbols. Scale bar, 20 μm. The data are expressed as the means ± SEMs. Green color (GFAP or Cx-43), blue color (DAPI), red color (Evans blue). * *p* < 0.05 was considered to indicate statistical significance. The white bar in A represents 20 μm.

Additionally, the penetration of Evans dye into the three tissues was significant for the cerebellum (*t* = 4.543, df = 4, *p* = 0.0105) of CTRL, with an average of 5.68 ± 0.64 SEM, and HFD-fed, with an average of 21.17 ± 3.35 SEM; the hippocampus (*t* = 4.295, df = 4, *p* = 0.0127) for CTRL, with an average of 5.94 ± 1.02 SEM, and HFD-fed, with an average of 11.96 ± 0.96 SEM; and the hypothalamus (*t* = 4.212, df = 4, *p* = 0.0136) for CTRL, with an average of 7.67± 2.06 SEM, and HFD-fed, with an average of 26.51 ± 3.98 SEM. We observed differences in the intensity of GFAP (Figure 5) across regions; greater intensity was observed in the cerebellum (Figure 5A vs. D; *t* = 10.65, df = 16, *p* < 0.0001), hippocampus (Figure 5B vs. E; *t* = 5.867, df = 14, *p* < 0.0001), and hypothalamus (Figure 5C vs. F; *t* = 7.964, df = 16, *p* < 0.0004) in the CTRL group than in the HFD-fed group. The increase in Evans dye penetration and GFAP intensity across the cerebellum, hippocampus, and hypothalamus in HFD-fed animals underscores the impact of an HFD on BBB integrity and astrocyte activation.

The intensity also differed across regions for CX43 (Figure 5), with greater intensities for the CTRL group than for the HFD-fed group in the cerebellum (Figure 5H vs. K; *t* = 72.04, df = 16, *p*< 0.0001), hippocampus (Figure 5I vs. L; *t* = 20.66, df = 16, *p* < 0.0001), and hypothalamus (Figure 5J vs. M; *t* = 32.92, df = 16, *p* < 0.0001).

When we evaluated the ultrastructural level in those areas, we observed that in the cerebellum (Supplementary Figure S1), the endothelial cells of the blood vessel next to the Purkinje cell bodies had a similar thickness around the contour of the vessel, and the capillaries presented a continuous morphology. In HFD-fed mice, the endothelium of the blood vessels of the cerebellar cortex showed remarkable homogeneous thinning and capillaries, with thinning of endothelial cells and a loss of continuous and resembling fenestrated capillaries.

**Figure 6:**
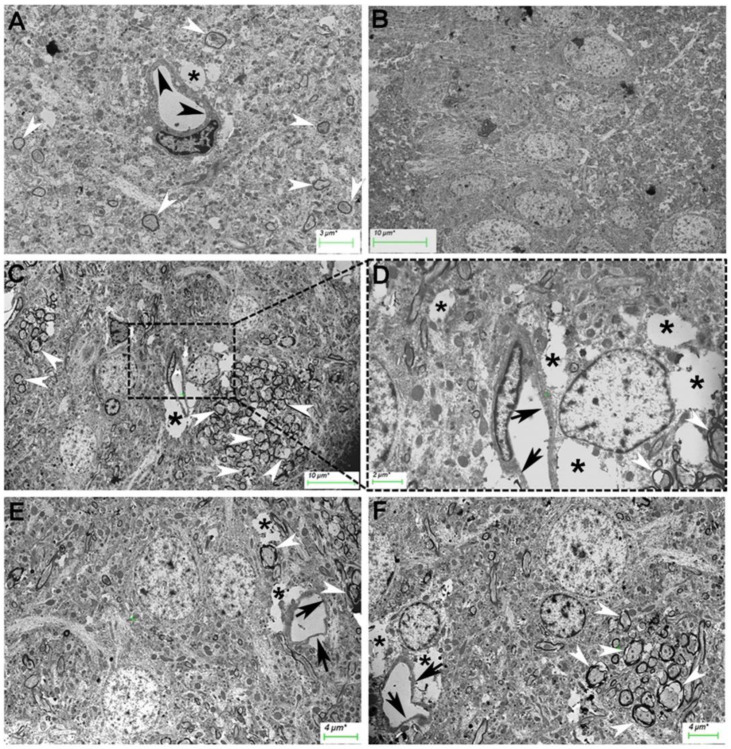
Transmission electron micrographs of hippocampi from normal rats (**A**,**B**) and HFD-fed rats (**C**–**F**). Hippocampal capillaries with homogeneous endothelial thickness (**A**, black arrowheads), neuropil (**B**), and myelin sheath (**A**, white arrowheads). Some small electrode spaces were occasionally observed near the blood capillaries (**A**, asterisk). HFD-fed rats (**C**–**F**). Capillaries (Caps) were observed with an irregular profile and were partially surrounded by clear spaces (asterisks). Spaces and apparent ruptures of axonal integrity (arrows). Spaces at the neuropil were also observed (**B**,**C**).

Hippocampal capillaries from normal rats presented characteristics of continuous capillaries with homogeneous endothelial thickness (Figure 6). The main ultrastructural alteration observed in HFD-fed rats was the thinning of the capillary endothelium, many of which had numerous electron-lucent spaces, most likely occupied by interstitial fluid present in the neuropil. No ultrastructural differences in neuronal nuclei were observed between the CTRL and HFD-fed groups.

**Figure 7:**
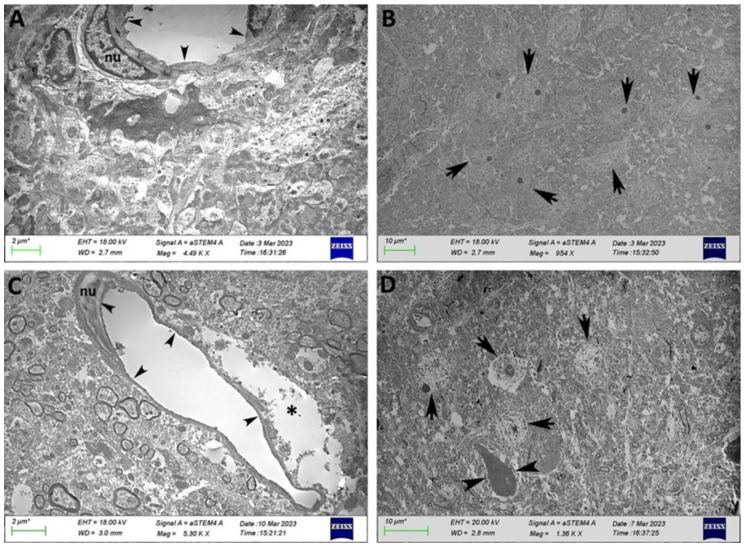
Transmission electron micrograph of the hypothalamus from a normal rat (**A**,**B**) and an HFD-fed rat (**C**,**D**). Capillaries (**A**, arrowheads; nu: nucleus). Endothelial cells (**C**, arrowheads). Nuclear chromatin condensation pattern (**C**, nu). Tissue fluid (**C**, asterisk). Nuclei with prominent nucleoli in the normal rat (**B**, arrows). Swelling nuclei, absent nucleoli, and karyolysis in the HFD-fed rat (**D**, arrows). Some neurons were highly dense in electrons (**D**, arrowheads).

Finally, regarding the capillaries in the hypothalamus (Figure 7), we observed the morphology of continuous capillaries and nuclei with abundant euchromatin and heterochromatin associated with the nuclear envelope (Figure 7A). In HFD-fed mice, the thickness of the cytoplasm of endothelial cells decreased (Figure 7C), and alterations in the nuclear chromatin condensation pattern were detected (Figure 7C). Some capillaries in these animals were partially surrounded by clear spaces, which were probably occupied by tissue fluid (Figure 7C). Numerous neurons presented nuclei with prominent nucleoli in normal rats (Figure 7B), whereas the neurons from HFD-fed rats presented swollen nuclei and no nucleoli or karyolysis (Figure 7D). Some neurons were highly dense in electrons (Figure 7D). HFD consumption led to alterations in capillary and neuronal morphology in the cerebellum, hippocampus, and hypothalamus, including decreased endothelial cell cytoplasm thickness, changes in nuclear chromatin condensation, and signs of neuronal damage.

## 3. Discussion

In this study, we aimed to investigate the structure–function relationship between allodynia and motor impairment in rats fed an HFD under conditions of obesity and MetS. We demonstrated that, within a short period (8 weeks), an HFD (60% of daily intake) leads to significant changes in metabolism and triggers the development of MetS, with impacts on allodynia and motor activity, as well as changes in the BBB, GFAP, and CX43 intensity and ultrastructural modifications in key brain regions, including the hippocampus, cerebellum, and hypothalamus. This highlights the profound impact of an HFD on both the peripheral and central nervous systems.

Our results align with observations from other research groups who reported that a high-fat commercial diet [27] induces obesity [28,29,30]. An interesting precedent to this work is the findings of Buettner and colleagues, who reported that an HFD, including lard and olive oil, significantly increased the weight of rats compared with that of CTRL [29]. This finding is reasonable because of the caloric content of fats (1 g of fat equals 9 kcal). In our study, the weight of the animals increased despite decreased food intake (Figure 1). Additionally, the effects of an HFD on the development of obesity depend on the duration of treatment, ranging from 4 to 40 weeks, and the percentage of calories from fat, ranging from 37% to 60% [31]. Hu et al. [32] revealed that after four weeks of consuming an HFD (with 60% of calories from lipids), the body weights of the animals did not significantly differ between groups. However, in our results, which were obtained via an 8-week HFD (60%), the change in weight gain began at four weeks. This change is consistent with previous findings and the study by Hu et al., where rats fed an HFD accumulated more adipose tissue in the gonadal and visceral regions. Our data confirm that the duration and fat content of an HFD are critical factors in the development of obesity, with significant weight gain observed as early as four weeks. These findings align with those of previous studies and underscore the role of HFDs in adipose tissue accumulation and obesity onset.

Saturated fat consumption induces insulin resistance and other metabolic defects, whereas monounsaturated fat consumption is considered protective against cardiovascular and metabolic alterations. However, previous studies have shown that olive oil or lard have nearly similar metabolic effects when they are used in high percentages [33]. Indeed, chronic, long-term (11 weeks) high-fat intake may subsequently promote the development of MetS [34,35]. Although we anticipated increased liver lipid accumulation, our acute 8-week exposure model did not produce significant changes in this parameter. However, metabolic alterations were evident, as indicated by the significant increase in HOMA–IR, reflecting impaired insulin sensitivity associated with HFD.

Previous studies have suggested that mechanical stress affects skeletal tissues, leading to chronic and painful disorders [36], and on the basis of the results obtained in our study, we propose that overweight status is a significant contributor to pain. Specifically, hypersensitivity to mechanical stimulation is a typical sign of neuropathic pain [37]; a study showed that obese animals fed a Western diet (high fat, high sucrose, high cholesterol) for eight weeks experienced this type of pain [22]. Our findings revealed that an 8-week HFD resulted in mechanical allodynia, with sensitivity to von Frey filaments approximately 40% greater than that in CTRL rats. Compared with the CTRL group, the HFD-fed group of Sprague–Dawley rats presented a lower mechanical threshold after eight weeks (significantly lower after ten weeks), which persisted after 12 weeks [23]. Numerous studies have examined the nociceptive response to mechanical allodynia in mice of different C57BL/6 strains and at various feeding durations (6, 7, 8, and 16 weeks), and a significant reduction in the mechanical threshold was observed [38,39,40]. According to Guillford and colleagues, mechanical sensitivity increased in nondiabetic C57BL/6 mice after eight weeks on an HFD compared with that in CTRL C57BL/6 mice [41], aligned with our model.

In addition, the accelerating or constant-speed rotarod test is commonly used to diagnose neurological injuries through motor coordination impairment [42]. As performing a task requires both kinematic (spatial direction) and kinetic (force) bodily movements, motor coordination is needed [24]. In our research, rats fed an HFD for eight weeks presented greater neurological and motor coordination impairments than did rats fed a healthy diet. This decline in motor coordination has been observed as early as four weeks in Wistar rats [24], 5 weeks in C57BL/6J mice [43], 12 weeks in Wistar rats [44], and 12 weeks [25] to 22 weeks [26] in C57BL/6J mice. Our research aligns with previous studies demonstrating that an HFD leads to motor coordination impairments, as evidenced by the rotarod test, reinforcing the utility of this test in diagnosing neurological deficits associated with metabolic disturbances.

In this study, we demonstrated that physiological changes, such as allodynia and motor alterations, are closely associated with morphological changes in BBB permeability and vary according to extrahypothalamic structure (see Figure 5). In this context, we detected differences when we analyzed the cerebellum, hippocampus, and hypothalamus. These differences are so complex that the close relationships between the major cellular components of the BBB, such as endothelial cells and astrocytic cells, must be understood. Our GFAP data align with those of Valdez and colleagues [45], who reported the absence of reactive microglia in the hypothalamus, suggesting that there was no neuroinflammatory response in animals exposed to an olive oil-rich diet. However, similar to the results of a study by Severi and colleagues [18], our ultrastructural data revealed thinning of the capillary endothelium in HFD-fed rats, many of which had numerous electron-lucent spaces in the hypothalamus. In our study, we observed similar changes in the hippocampus, and previous studies in animal models have shown that HFDs induce neuroinflammation in the hippocampus, which is associated with pain-related behaviors [46,47]. Our model highlights the relationships between physiological changes, such as allodynia and motor alterations, and morphological changes in BBB permeability across different brain structures. These results underscore the importance of understanding the interactions between BBB components, such as endothelial and astrocytic cells, in the context of diet-induced neuroinflammation and its impact on neurological functions.

Recent research on the hippocampus of adult offspring revealed a positive correlation between GFAP and the gap junction protein CX43 in response to an HFD [12]. In our study, the observed decrease in GFAP intensity was associated with a decrease in CX43, including in the hippocampus, cerebellum, and hypothalamus. However, ultrastructural analysis reveals an irregular profile and is partially surrounded by clear spaces such as those described previously in the median eminence [18], hippocampus [48], and cerebellum [19]; these morphological changes could explain the alterations in functional tests.

While our ultrastructural analysis provided valuable insights, it was limited to specific brain regions, leaving the potential effects on other regions unexplored. The partial decrease in GFAP and CX43 expression observed only in the hippocampus, cerebellum, and hypothalamus may reflect region-specific responses. Despite these limitations, the findings of this study are robust, are aligned with the literature, and provide consistent data on HFD-induced alterations in the structural and functional integrity of the brain. Expanding the ultrastructural analysis to include other brain regions and incorporating a broader array of neuroinflammatory markers could provide a comprehensive understanding of the brain’s response to an HFD.

## 4. Materials and Methods

### 4.1. Animals and general housing conditions

Adult male Wistar rats weighing 250–300 g were obtained from the vivarium of the School of Medicine at Universidad Nacional Autónoma de México. For acclimatization, the animals were housed in pairs and maintained in a soundproof monitoring room with a light/dark (LD) cycle (lights on at 07:00 h). The space was maintained at 22 ± 1 °C, 50% humidity, and continuous airflow. The rats had free access to food with standard chow (Laboratory Rodent Diet 5001, LabDiet^®^; Minneapolis, MN, USA) and water. The testing procedures used in this study were in strict accordance with the Mexican norms for animal handling (Norma Official Mexicana NOM-062-ZOO-1999), which conforms to international guidelines for animal handling and is approved by the Ethics Committee of the Medicine School UNAM (FMED/DI/067/2019 06082019). Furthermore, all efforts were made to minimize the number of animals and their suffering. Human endpoints were established for this study (Supplementary Table S1).

### 4.2. Establishing a Model of Obesity

#### 4.2.1. HFD

The HFD was prepared with the control diet as a base, adding fats of animal and vegetable origins. The preparation of the HFD [49] (100 g) consisted of pulverizing Laboratory Rodent Diet 5001 pellets (50 g) and adding lard (17.5 g), olive oil (17.5 g), and lyophilized egg albumin (15 g). Fatty acid analysis of the obtained paste was performed (Supplementary Table S2).

#### 4.2.2. Experimental Design

A total of 36 rats (the experimental unit) were used for this study. By simple randomization, the rats were randomly assigned to one of two conditions: the CTRL group (*n* = 18) or the HFD-fed group (*n* = 18). After two weeks of acclimatization with free access to food (Laboratory Rodent Diet 5001) and water, the rats in the experimental group were fed an HFD for eight weeks. Food consumption and weight were quantified with a digital weighing scale (Kent Scientific Corporation) each week throughout the testing period. One animal in each group was excluded from the analysis (Table 1) because it died during the insulin test.

#### 4.2.3. IPGTT and ITT

At the end of the eight weeks of treatment, eight animals in each group were fasted for 12 h to determine their FPG levels, and IPGTTs and ITTs were performed. For the IPGTT, 2 g of glucose/kg body weight was administered intraperitoneally; for the ITT, 0.2 IU of human recombinant insulin (Humulin, Lilly; Indianapolis, IN, USA) was administered intraperitoneally. Blood glucose was measured at 0, 30, 60, 90, and 120 min after the intervention via a handheld blood glucose monitor (OneTouch^®^ UltraMini^®^, Harrisburg, PA, USA).

#### 4.2.4. Insulin Resistance Surrogate Measures

Insulin resistance was estimated according to the HOMA–IR, which was previously adapted for use in Wistar rats [50]: HOMA–IR = (FPG (mg/dL) × FPI (µUI/mL))/2430.

Insulin concentrations (pmol/L) were converted for use in the above formula: 1 µUI/mL = 6 pmol/L [51].

#### 4.2.5. Plasma, Adipose Tissue, and Brain Samples

At the end of the eight weeks of treatment, 18 animals in each group were anesthetized with intraperitoneal (i.p.) sodium pentobarbital (Pisabental^®^, Aranda; 100 mg/kg; Mexico City, Mexico), and epidydimal and subcutaneous fat pads were obtained. The weight of the fat pads was reported as a proportion of the total body weight. Additionally, 5 mL of blood was drawn from the inferior vena cava into tubes with EDTA for 11 rats per group. To obtain plasma and measure glucose, leptin, and insulin concentrations, the blood was immediately centrifuged at 1800 rpm for 10 min at room temperature and subsequently frozen in 0.2 mL aliquots at −80 °C until analysis. The brain, liver, and adipose tissue were removed. Leptin and insulin levels were determined via a Milliplex^®^ MAP Rat Adipokine Magnetic Bead Panel (RADPKMAG-80K; Merck Millipore; Hesse, Germany) according to the manufacturer’s instructions.

#### 4.2.6. Histological Preparation

The brain and adipose tissue were washed in ice-cold phosphate-buffered saline (PBS), followed by the addition of 4% paraformaldehyde in PBS at 4 °C. After fixation, the tissues were dehydrated and embedded in paraffin for sectioning.

These tissues were analyzed via H&E-stained paraffin sections (cut into four-μm-thick sections) from both groups.

### 4.3. Behavioral Tests and Damage Induced by an HFD

#### 4.3.1. Mechanical Allodynia

Behavioral tests were performed in all the rats at the basal stage (day zero) and after 8 weeks in the CTRL (*n* = 6) and HFD-fed (*n* = 6) rats, according to a method published by Chaplan [52]. Von Frey filaments were used to stimulate the plantar aspect of the foot, and the 50% PWT was determined via the up–down method [53].

#### 4.3.2. Motor Coordination

All the rats were trained with a 5 min run at a steady speed of 4 rpm for 3 days before testing. The next day, the animals were placed in the rotating cylinder at a rotational speed that was increased from 4 rpm to 40 rpm for 5 min. The latency to fall was recorded up to a limit of 300 s [54].

### 4.4. Evaluating BBB Integrity

#### 4.4.1. Evans Blue Administration

After the experiment, three rats in each group were anesthetized via an intraperitoneal injection of 60 mg/kg sodium pentobarbital. A 2 cm thoracic incision was made to expose the thoracic cavity. The rats were then perfused intracardially with 250 mL of 0.9% saline solution, followed by 4% paraformaldehyde (PFA) prepared in a buffered 0.1 M sodium phosphate pH 7.2 (PB) solution plus Evans blue [55].

#### 4.4.2. Immunofluorescence

To conduct immunofluorescence, the extracted brains were postfixed in 4% PFA at 4 °C for 24 h. Afterward, they were cryoprotected in a 30% sucrose solution. The tissue was embedded in an optimal cutting temperature (O.C.T.) compound (Freeze Mount, Mount Vernon, WA) and processed under freezing conditions. A cryostat (Leica CM1100) was used to obtain four series of 20-micrometer-thick coronal sections. Sections were collected in two series: one series was processed for GFAP, and the other series was processed for CX43. Free-floating sections were blocked with 2% albumin, and the primary antibodies mouse anti-GFAP (1:100; PM065AA, BioCare Medical, California, CA) or anti-connexin 43 (F-7) (1:50; SC-271837, Santa Cruz, TX, USA) were added and incubated overnight at 4 °C. Each well was then washed four times for ten minutes with 1X PBS while agitated. The secondary antibody, goat anti-mouse IgG (1:100; 62-6511, Invitrogen, Massachusetts, MA, USA), was incubated at room temperature for two hours in the dark. DAPI-containing mounting medium was added (VECTASHIELD^®^, California, CA, USA). The cover slip, which was previously cleaned with gauze and alcohol, was placed on the slide 5 min later. The edge was protected by a transparent varnish coating. After microscopic examination, the samples were stored at 4 °C.

#### 4.4.3. Image Digitalization

The slides were examined via a Zeiss (LSM 880 Zeiss; Oberkochen, Germany) confocal laser scanning microscope, and the laser intensity and digital gain were held constant between groups. Using the ImageJ software version 1.46r (https://imagej.net/software/imagej/) package, the marker density, corrected fluorescence, morphological parameters of the cells and cell type were determined from the images.

#### 4.4.4. Transmission Electron Microscopy

Three rats (by group) were anesthetized i.p. with sodium pentobarbital (Pisabental^®^, Aranda; 100 mg/kg; Mexico City, Mexico), and their whole bodies were fixed via a gravity-fed perfusion apparatus with two bottles interconnected with three-way keys, one containing heparinized physiological saline solution (PSS) and the other containing 2.5% glutaraldehyde fixative solution in 0.1 M sodium cacodylate buffer (pH 7.4). This apparatus was placed approximately 130 cm above the animal [56]. A thoracotomy was carried out to insert a needle into the left ventricle of the heart. Afterward, a cut was made in the right atrium allowing the exit of blood, whereas the blood was removed from the circulatory system with the perfusion PSS for 20 min. When only PSS exited from the right atrium, the fixative solution was perfused for 20 min. Next, the rats were decapitated to remove the brains from the skull. Manual brain samples (1 mm^3^ each) containing the cerebellum cortex, hippocampus, and hypothalamus (near the III ventricle) were obtained and immersed in the same fixative for 3 h at 4 °C. The samples were then washed in the same buffer (0.2 M, pH 7.4) and postfixed in 1% osmium tetroxide diluted in the same buffer for 1 h at 4 °C. The samples were dehydrated in gradually increasing ethanol concentrations, transferred to propylene oxide, and embedded in an EPON 812 embedding medium. Thin 0.9-μm-thick sections obtained with a glass knife were stained with toluidine blue in borax to select the microscopic field from which ultrathin sections were cut with a diamond knife. Ultrathin sections were mounted on copper grids, contrasted with 2% aqueous uranyl acetate and 2% plumber citrate, and observed with a Zeiss STEM electron microscope (Crossbeam 550). Six ultrathin sections were analyzed for each CNS region.

#### 4.4.5. Data Analysis

GraphPad Prism software version 9 (GraphPad Software Inc., San Diego, CA, USA) was used for the statistical analyses. Descriptive statistics are presented as the mean ± standard error of the mean (S.E.M.) for the quantitative variables BMI, abdominal circumference, body length, Lee index, FPG, leptin, insulin, triacylglycerols, HOMA–IR, epididymal fat, subcutaneous fat, optical density of Evans blue, quantification of GFAP, and CX43 staining intensity. To quantify the global response of glucose and insulin in the blood after the test, an AUC analysis was performed. The data were tested for normality with the Shapiro–Wilk test. When suitable, differences between groups were assessed through unpaired *t*-tests or with Mann–Whitney U tests when the data did not meet the normality assumption.

Statistical differences between groups in terms of body weight gain, diet consumption, and IPGTT and ITT results were determined via repeated measures (RM) ANOVA followed by Sidak’s multiple comparisons test or one- or two-way ANOVA followed by Tukey’s test or the Kruskal–Wallis test followed by the Dunn test for variables that were not normally distributed.

A sample size calculation was performed to detect a 75% difference in altered behavior proportions between the CTRL and the HFD-fed group, with 95% confidence and 80% power, yielding a sample size of 6. Mechanical allodynia and motor coordination data are presented as the means ± S.E.M.s of 6 independent animals. Between-group differences were assessed via one-way analysis of variance (ANOVA) or the Kruskal–Wallis test, followed by the Mann–Whitney U test. A *p*-value < 0.05 was considered to indicate statistical significance.

## Figures and Tables

**Table 1:** Effects of diet on somatometric and physiological parameters after 8 weeks.

Parameters	Groups		*p*
	CTRL	HFD-Fed	
BMI (kg/m^2^)	0.67 ± 0.02	0.79 ± 0.03	**0.002** *
Abdominal circumference (cm)	19.09 ± 0.13	21.23 ± 0.28	**0.001** *
Body length (cm)	25.86 ± 0.35	24.50 ± 0.44	**0.026** *
Lee index (g/cm)	295.34 ± 4.42	318.70 ± 5.68	**0.004** *
Fasting plasma glucose (mg/dL)	81 ± 1.58	91 ± 4.54	**0.022** *
Leptin (pg/mL)	26,547.19 ± 7785	58,447.19 ± 9227	**0.007** *
Insulin (pg/mL)	3180.77 ± 366.2	4621.67 ± 691.1	**0.042** *
Triacylglycerols (mg/dL)	99.11 ± 4.47	118.16 ± 18.39	0.177
HOMA–IR (%)	18.38 ± 1.86	31.76 ± 4.59	**0.012** *

Bold numbers indicate significant differences. The data are expressed as the means ± SEMs (*n* = 11 rats). * *p* < 0.05 was considered to indicate statistical significance.

## Data Availability

Data that support the findings of this study are available from the corresponding author upon reasonable request.

## Supplementary Materials

The following supporting information can be downloaded at https://www.mdpi.com/article/10.3390/ijms252011218/s1.
