# Supplementary material for: High-Fat Diet-Induced Blood–Brain Barrier Dysfunction: Impact on Allodynia and Motor Coordination in Rats
Source: Int J Mol Sci. 2024 Oct 18;25(20):11218. doi: 10.3390/ijms252011218 (PMC11508281; doi:10.3390/ijms252011218)
Supplement: Supplementary file 1 [file ijms-25-11218-s001.zip › Supplementary Tables S1 and S2_IJMS.pdf]

Table S1. Guidelines Human Endpoints

Supervision Protocol		
	Degrees	Points
Appearance	Normal	0
	Moderate	1
	Severe	2
Grimace Scale	Not present	0
	Moderately present	1
	Obviously present	2
Locomotion	Normal	0
	Moderate	1
	Diminish	2
Weight	Normal	0
	Moderate (lost 10%)	1
	Severe (lost 15%)	2
Faecal/urine	Normal	0
	Moderate	1
	Zero	2
	Total points	0-10

Interpretation	
Total score	Overall assessment
0-2	Normal
3-5	Monitor carefully
6-8	Analgesic meloxicam 1.5mg/kg, SC/12h/3 days
9-10	Terminate the experiment, pentobarbital 120mg/kg IP

Notes:
SC subcutaneous
IP intraperitoneal

Table S2. Fatty acid composition of the High Fat Diet

Fatty acids	Composition, g/100 g
Saturated fatty acids (SFA)	
Caprilic acid (8:0)	0.002
Capric acid (10:0)	0.025
Lauric acid (12:0)	0.015
Myristic acid (14:0)	0.247
Pentadecanoic acid (15:0)	0.013
Palmitic acid (16:0)	6.550
Margaric acid (17:0)	0.067
Stearic acid (18:0)	2.323
Heneicosanoic acid (21:0)	0.152
Behenic acid (22:0)	0.090
Lignoceric acid (24:0)	0.004
Total	9.488
Unsaturated fatty acids (UFA)	
Miristoleic acid (14:1)	0.030
Palmitoleic acid (16:1)	0.814
cis-10-Heptadecenoic acid (17:1)	0.076
Oleic acid (18:1)	19.153
cis-11-Eicosenoic acid (20:1)	0.306
Erucic acid (22:1)	0.002
Lignoceroleic acid (24:1)	0.014
Total monounsaturated fatty acids	20.413
Linoleic acid (18:2)	4.677
γ Linolenic acid (18:3)	0.101
Linolenic acid (18:3)	1.020
Eicosadienoic acid (20:2)	0.128
cis-8,11,14-Eicosatrianoic acid (20:3)	0.013
cis-11,14,17-Eicotrianoic acid (20:3)	0.025
cis-13,16-Docosadienoic acid (22:2)	0.022
Total polyunsaturated fatty acids	5.986
Total fatty acid content	35.887
