# Supplementary figures and images for: High-Fat Diet-Induced Blood–Brain Barrier Dysfunction: Impact on Allodynia and Motor Coordination in Rats
Source: Int J Mol Sci. 2024 Oct 18;25(20):11218. doi: 10.3390/ijms252011218 (PMC11508281; doi:10.3390/ijms252011218)

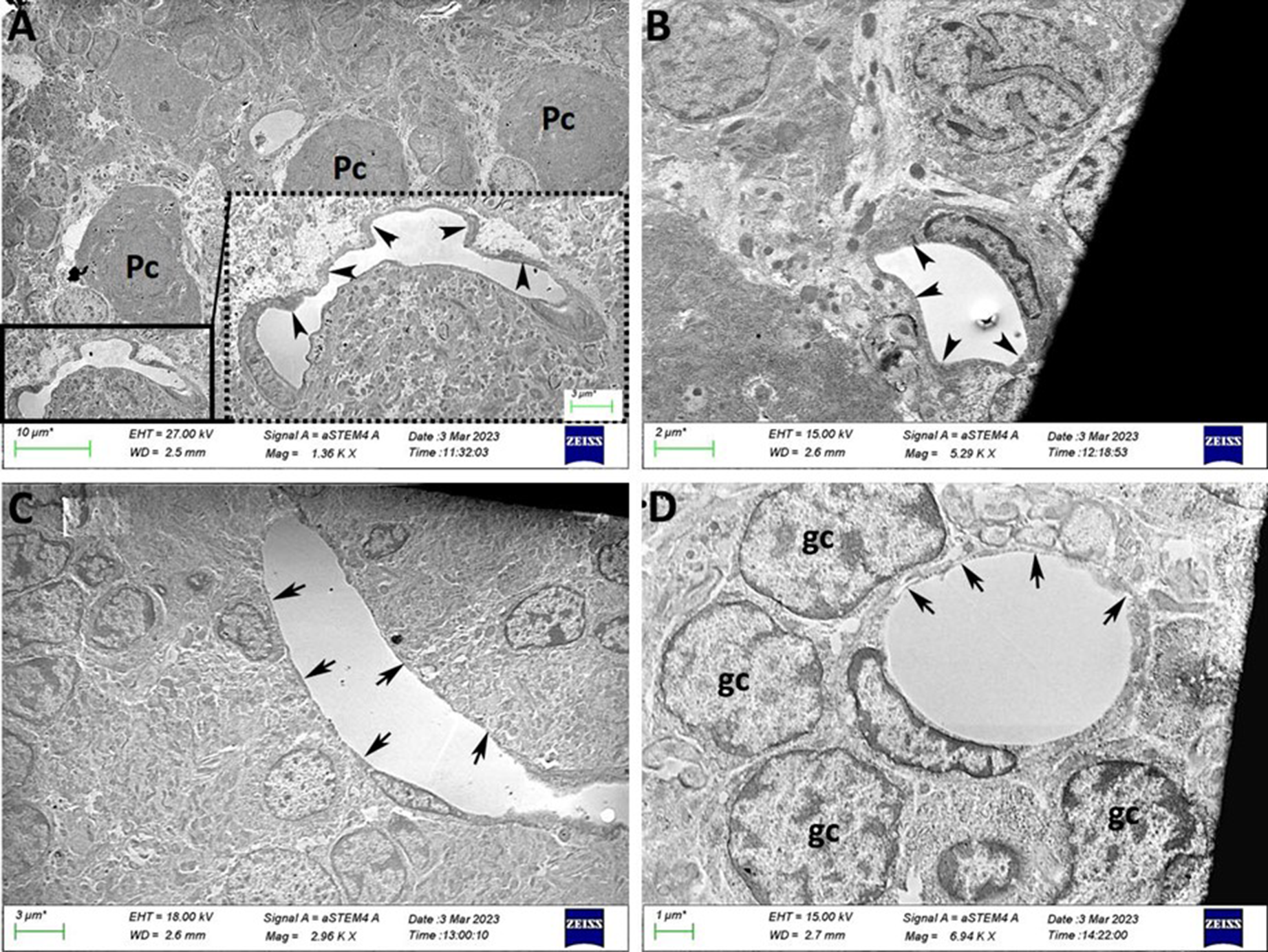

Supplement: Supplementary file 1 [file ijms-25-11218-s001.zip › Supplementary Figure S1.tif]
